# Prenatal diagnosis of microcephaly through combined MRI and ultrasonography: Analysis of a case series

**DOI:** 10.1097/MD.0000000000036623

**Published:** 2023-12-15

**Authors:** Shu-ping Pei, Hai-lian Guan, Feng Jin

**Affiliations:** a Department of Obstetrics, Tongde hospital of Zhejiang province, Hangzhou, China.

**Keywords:** fetus, microcephaly, MRI, US

## Abstract

**Introduction:**

Intrauterine microcephaly is a complex and lifelong condition that poses significant ethical challenges for clinicians and parents. The prognosis of microcephaly is highly variable and depends on the underlying cause and severity. In addition, microcephaly is often associated with various comorbidities, including intellectual disability, developmental delay, and epilepsy. Ultrasonography (US) is currently the most commonly used imaging modality for detecting microcephaly in the second trimester of pregnancy. However, antenatal brain magnetic resonance imaging (MRI) is increasingly being used as a more sensitive tool to identify structural abnormalities that may suggest a specific diagnosis. In this study, we report a case series of microcephaly diagnosed through the combination of MRI and US.

**Patient concerns:**

How to utilize a combination of MRI and US to screen for fetal microcephaly.

**Diagnosis:**

Based on the results of US and MRI examinations, patient 1 was found to have other craniocerebral malformations, patient 2 demonstrated macrogyria, and patient 3 exhibited skull irregularities.

**Interventions:**

The pregnancies of all 3 patients were terminated through the induction of labor by injecting Rivanol into the amniotic cavity.

**Outcomes:**

The 3 patients were discharged after a period of observation.

**Conclusion:**

US is an important tool for diagnosing fetal microcephaly. However, MRI can overcome the limitations of US and detect additional brain structural abnormalities, thereby providing more specific and valuable prenatal diagnostic information. Therefore, combining MRI and US has significant diagnostic value for fetal microcephaly.

## 1. Introduction

Microcephaly is diagnosed when the occipital frontal head circumference (OFC) is 2 standard deviations (SD) less than the expected average for age, gender, and population.^[1]^ Severe microcephaly is defined as an OFC that is more than 3 SD below the average.^[1]^ The fetal head circumference reflects the brain volume, making it an essential indicator for detecting fetal brain development. The presence of microcephaly suggests an abnormal brain development that can lead to various neurological sequelae, including epilepsy, cerebral palsy, and intellectual impairment. However, a small head circumference is not always indicative of poor nervous system development. Therefore, early prenatal diagnosis of microcephaly is essential for appropriate clinical intervention. Currently, traditional prenatal ultrasonography (US) is the primary diagnostic method for microcephaly, while magnetic resonance imaging (MRI) has shown promise in the diagnosis of fetal microcephaly, although experience with this technique is still limited, and there is a need for standardized understanding of this disease. In this report, we present brain US and MRI images of 3 patients with microcephaly to explore the potential diagnostic value of combining MRI and US in the diagnosis of fetal microcephaly.

## 2. Methods

The following report delineates 3 successive cases of Microcephaly, managed by an obstetrician within an 8-month span in 2022 at Tongde Hospital of Zhejiang province. Microcephaly diagnoses were confirmed through perinatal MRI procedures (with gestational ages exceeding 24 weeks) and US, as documented in medical records. Comprehensive data were harvested from each case previous prenatal ultrasound examinations.

Experienced, certified physicians conducted all ultrasound examinations utilizing the GE Kretz Voluson E8/E10 and a 4 to 8 MHz transabdominal probe. In adherence to the stipulations of the “Guidelines for Prenatal Ultrasound Examination (2012)” and the “ISUOG Guidelines for Ultrasound of the Fetal Central Nervous System,” the ultrasound image collection was completed.

Each examination encompassed a scan of the overall fetal condition, assessing for coexistent extracranial deformities, and providing a meticulous examination of brain structures across various planes. This included measurements and image preservation of the parietooccipital sulcus, talus sulcus, and lateral fissure. For the dual purposes of training and quality control, a supervisor retrospectively reviewed all cases. Patient data were anonymized and securely stored in an electronic database.

The Tongde Hospital of Zhejiang Province Ethics Commission for human research sanctioned the study, ensuring the ethical standards and integrity of the research were maintained, and all patients gave informed consent.

## 3. Results/case description

### 3.1. Case 1

**Figure 1.**
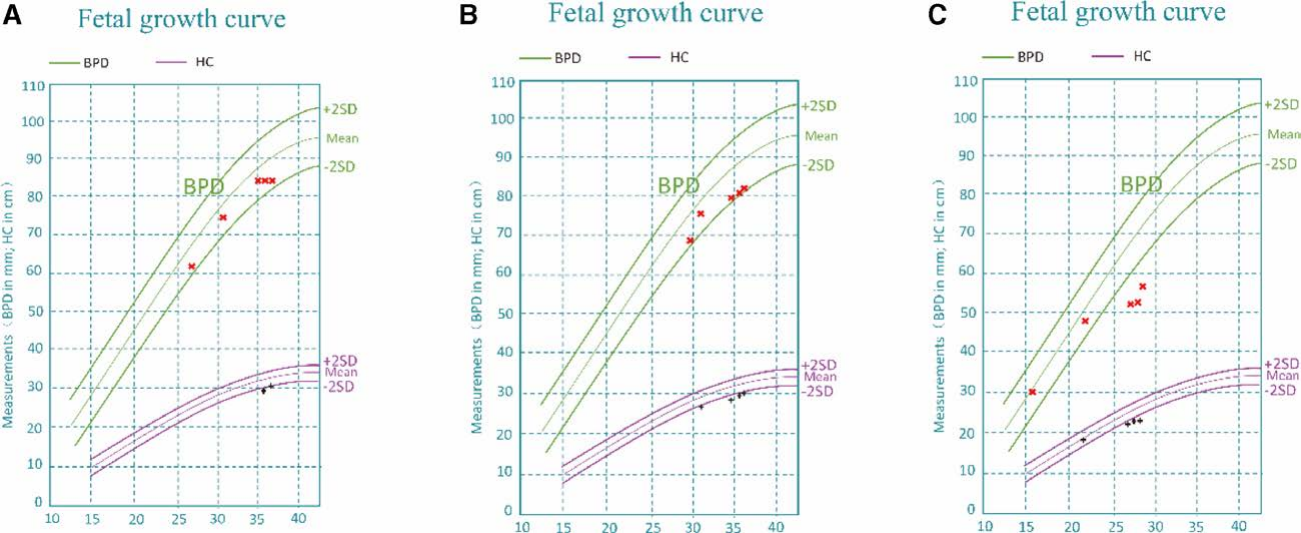
BPD and HC growth chart. The magnitude of BPD and HC measured by ultrasound was plotted using the x-labels. BPD = biparietal diameter, HC = head circumference.

**Figure 2.**
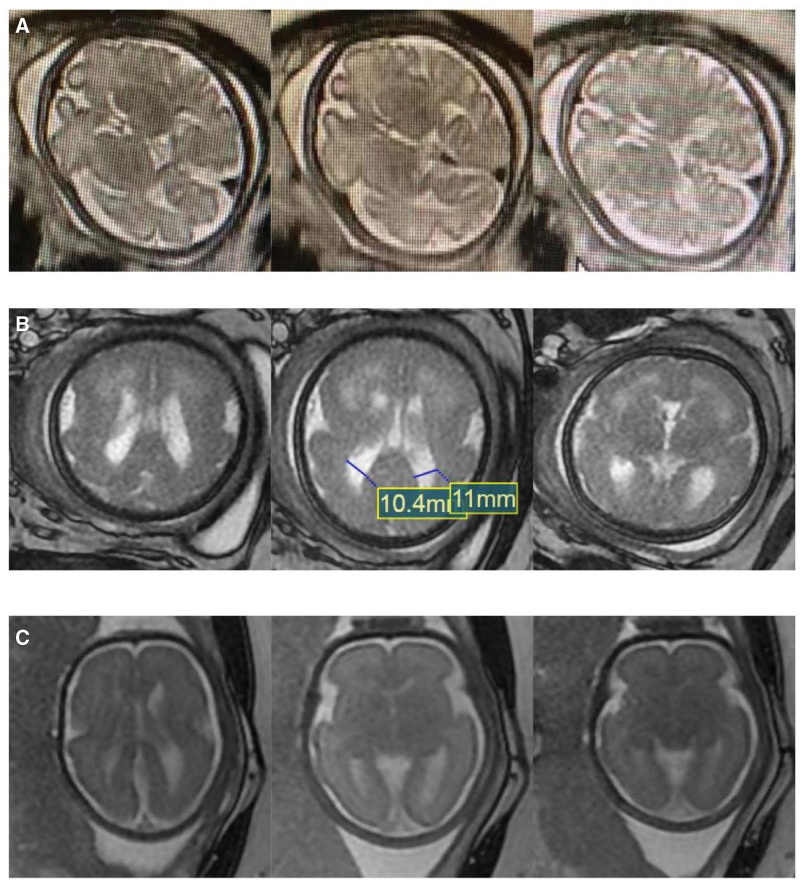
Prenatal fetal MRI findings. (A) Thinning of the white matter in the temporal and frontal parietal areas of both sides of the brain, as well as patchy shadows under the frontal lobe cortex on both sides. Compared to a normal fetus at 37 wks of gestation, it was determined that the white matter in the brain of the fetus was dysplastic. (B) The brain contour exhibited the “8” character sign, and a curved T2 high signal shadow was observed in the frontal parietal cortex. The width of the posterior horn of the left and right ventricles was approximately 11 and 10.4 cm, respectively, with a transparent compartment cavity present. No obvious widening was observed in the cisterna magna, and the cerebellar vermis was visible. (C) A normal signal. MRI = magnetic resonance imaging.

A 30-year-old Chinese woman presented with a complaint of no significant increase in fetal biparietal diameter (BPD) for 3 consecutive weeks during the third trimester of her pregnancy. The patient had normal prenatal screening results after starting routine antenatal examinations, with no apparent unusual circumstances. However, since 35 + 1 weeks of gestation, there had been no increase in the BPD of the fetus. In the obstetrical department, a gynecological US examination was performed which revealed the following: there was no significant increase in fetal BPD for 3 consecutive weeks from 35 to 37 weeks of gestation. Fetal head circumference was <2 SD for the gestational age since 35 weeks of gestation. The fetus did not have any deformities (see Fig. 1A for details). According to the results of her physical examination and US, the fetus was suspected to have microcephaly. In order to confirm the diagnosis and identify the cause of microcephaly, a fetal MRI scan was performed at 37 weeks of gestation, and the risks and benefits were explained to the patient and her family. The fetal MRI scan revealed thinning of the white matter in the temporal, frontal and parietal areas of both sides of the brain, as well as patchy shadows under the frontal lobe cortex on both sides. Compared to a normal fetus at 37 weeks of gestation, it was determined that the white matter in the brain of the fetus was dysplastic (Fig. 2A). Notably, the patient had no history of fetal malformations. The patient had no significant personal or family medical history. The changes in uterine height and abdominal circumference throughout pregnancy are presented normally. Conventional prenatal screening and routine blood tests were conducted before the fetal MRI examination, and the results were normal. Based on the results of US and MRI examinations, the patient was diagnosed with microcephaly. She was terminated through the induction of labor by injecting Rivanol into the amniotic cavity. Based on fetal chromosomal microarray analysis, the patient fetus shows a 491 Kb deletion in the 2q24.2 region. Additionally, 2 potentially pathogenic variants of the ASNS gene were detected in both the patient and their spouse peripheral blood samples. The clinical presentation of microcephaly and cortical abnormalities align with the autosomal recessive genetic disease Asparagine Synthase Deficiency (OMIM: 615574).

### 3.2. Case 2

A 26-year-old Chinese woman presented to the obstetrical department with complaints of a worsening fetal development trend during 31 to 34 weeks of gestation. The patient underwent normal prenatal screening with no abnormalities detected. However, during her visit to the hospital at 34 + 3 weeks of gestation, fetal US was conducted which revealed that both fetal BPD and head circumference were 2 to 3 SD smaller than expected. As a result, she was referred to our obstetrical department for further treatment, a follow-up 2-dimensional (2D) US was performed, which showed a further decrease in head circumference to <3 SD of gestational age, accompanied by oligohydramnios (AFI 7.3 cm) at 36 + 3 weeks of gestation (as summarized in Fig. 1B). Fetal MRI was also performed. MRI of the fetal brain revealed a smooth surface of bilateral cerebral hemispheres with wide gyri. The bilateral lateral fissure was shallow, asymmetrical, and vertical. The brain contour exhibited the “8” character sign, and a curved T2 high signal shadow was observed in the frontal and parietal cortex. The width of the posterior horn of the left and right ventricles was approximately 11 and 10.4 cm, respectively, with a transparent compartment cavity present. No obvious widening was observed in the cisterna magna, and the cerebellar vermis was visible (Fig. 2B). The patient had no significant personal or family medical history. She received pre-pregnancy vaccination against coronavirus. The physical examination showed no remarkable results, and there were no special obstetric conditions found. Routine blood tests and conventional immune indices examinations were reportedly normal before the fetal MRI examination. She was also diagnosed with microcephaly, along with the additional diagnosis of macrogyria. The patient was terminated through the induction of labor by injecting Rivanol into the amniotic cavity. The Patient fetus displayed no chromosomal abnormalities, and chromosomal microarray analysis did not detect any copy number variations >100 kb with pathogenic or potentially pathogenic consequences.

### 3.3. Case 3

A 26-year-old Chinese woman reported a discrepancy of 4 SD in head circumference at 27 weeks of gestation. The patient began receiving regular prenatal examinations starting at the 12th week of gestation, and the first trimester US screening was unremarkable. The 2D US examination showed normal fetal status, while the 3-dimensional (3D) US examination indicated that the fetus was 2 weeks behind the gestational age at 22 weeks of gestation. The fetus’ overall growth and development started to deteriorate, with a fetal head circumference measuring <4 SD at 27 weeks of gestational age, accompanied by a low amniotic fluid index (AFI) of 5.8 cm. (refer to Fig. 1C). Cerebral MRI at 28 weeks of gestation, exhibited a normal signal (Fig. 2C). The patient prenatal screenings did not indicate any significant abnormalities, and there was no history of fetal malformations. The patient had no significant personal or family medical history. She received pre-pregnancy vaccination against coronavirus. The fundal height was significantly smaller than expected for the gestational age. Routine blood examination was normal, including TORCH virus and immune index. The Patient was diagnosed with severe fetal growth restriction as well as microcephaly. She was terminated through the induction of labor by injecting Rivanol into the amniotic cavity. Her fetus did not undergo chromosomal examination.

## 4. Discussion

### 4.1. Diagnosis

Intrauterine microcephaly is a medical condition characterized by an abnormally small head circumference (occipito-frontal diameter) of a fetus, which is either equal to or smaller than 3 SD from the median head circumference for a given age, sex, and race, or <3 SD from the median head circumference for gestational age.^[1]^ This condition serves as a clinical indicator of abnormal brain development. Some scholars believe^[2]^ that a diagnosis of fetal microcephaly can be excluded when the fetal head circumference is greater than or equal to 2 SD, while a head circumference <5 SD can be diagnosed as pathological fetal microcephaly. When the head circumference is between 2 SD and 5 SD, pathological fetal microcephaly cannot be ruled out, and follow-up monitoring is necessary. According to the American Maternal-Fetal Medicine Society,^[3]^ a head circumference <3 SD can be diagnosed as isolated fetal microcephaly, while a head circumference <5 SD can be diagnosed as pathological fetal microcephaly, which necessitates detailed neurological US and periodic review. Currently, a head circumference <3 SD is widely accepted as a diagnostic criterion for fetal microcephaly due to its strong correlation with intellectual developmental disorders. Therefore, guidelines recommend establishing a diagnosis of fetal microcephaly when the fetal head circumference is <3 SD below the mean, along with conducting comprehensive and detailed examinations of fetal anatomical structure and attending to fetal brain examination results, intrauterine infections, potential teratogenic exposures, and extrencephalic symptoms related to chromosomal abnormalities or monogenic diseases.

Therefore, guidelines recommend establishing the diagnosis of fetal microcephaly when the fetal head circumference is <3 SD below the mean. A comprehensive and detailed examination of fetal anatomical structure is required, with attention paid to fetal brain examination results, intrauterine infections, potential teratogenic exposure, and extracephalic symptoms related to chromosomal abnormalities or monogenic diseases.

### 4.2. Prenatal US findings

In prenatal US diagnosis, the measurement of fetal head circumference is considered the most reliable indicator, surpassing single measurements obtained through ultrasonic examination. To evaluate the fetal head, a set of 4 standard views in the axial plane are typically assessed, including the falx view, trans-ventricular view, cavum view, and posterior fossa/trans-cerebellar views. The falx view, which is characterized by an echogenic and linear structure positioned between the cerebral hemispheres, allows one to see the falx cerebri. However, a trans-ventricular image obtained at the cavum septum pellucidum level is useful for detecting the septum that divides the lateral ventricles and determining if there is ventriculomegaly.^[4]^ When the transducer is positioned at the level of the midbrain and directed in the direction of the occiput to generate the posterior fossa picture, the cisterna magna, cerebellar hemispheres, and cerebellar vermis are all plainly visible.^[4]^ At the thalamic level, the cavum view is finally captured, exposing the cavum septum pellucidum as a rectangular formation positioned between the frontal horns of the lateral ventricles.^[4]^ The cavum view provides the most accurate assessment of microcephaly among the several fetal views investigated, allowing measurement of both BPD and OFC, also known as head circumference. These measurements are typically taken as part of the second-trimester ultrasound examination fetal biometric assessment. Electronic calipers are placed perpendicular to the falx cerebri at the broadest part of the head in order to measure the BPD. The occipital-frontal diameter (OFD), as measured by calipers positioned at the middle of the echoic bone on both the frontal and occipital areas, is then used to determine the OFC. Then, to calculate the OFC (or head circumference), the values of BPD and OFD are entered into the formula OFC (or head circumference) = 1.62 × (BPD + OFD).^[5]^ The resulting measurement is then compared to standardized reference charts outlining the usual values of the OFC for a certain gestational age and gender.^[5]^

Microcephaly is often associated with abnormal development of cerebral gyri and sulci. Cerebral gyri are the most prominent features on the surface of the cerebral cortex, and their formation process is a clear manifestation of fetal brain maturation.^[6]^ Abnormal development of sulci may serve as an early warning sign of potential fetal neuronal migration disorders,^[7]^ which are often accompanied by abnormalities in cranial morphology, volume, central nervous system, and fetal chromosomes. To assess the development of the cortex, it is a common practice to evaluate fetal sulci development through prenatal US. In a seminal study, Toi et al^[8]^ examined 50 normal fetuses between 15 and 29 weeks of gestation and identified the parietal-occipital sulci, taloccipital sulci, cingulate gyri, and cerebral gyri at 20, 21, 24, and 27 weeks of gestation, respectively. This study established a critical milestone for the normal development of the cerebral gyri and enabled the early detection of cortical abnormalities through 2D US. Domestic scholars conducted cross-sectional studies to observe the normal development of the fetal insula, lateral fissure, parieto-occipital sulcus, and temporo-occipital sulcus from 18 to 41 weeks of gestation. Their findings revealed that the lateral fissure width, temporal lobe depth, parieto-occipital sulcus depth, and temporo-occipital sulcus depth increased with gestational age, while the uncovered insula width and parieto-occipital sulcus angle decreased with gestational development.^[9]^ Fetal sulci and gyri development are delayed by more than 3 weeks compared to normal fetuses of the same gestational age, and there is no obvious catch-up phenomenon in dynamic observation. This delay is referred to as sulci and gyri developmental retardation. It is important to note that only a portion of the sulci can be detected by 2D abdominal US, and an MRI may be necessary for a complete diagnosis.

Additionally, during US, attention should be paid to the observation of craniosynostosis, which can often be caused by brain development problems in microcephaly. About 2% of microcephaly cases are associated with craniosynostosis,^[10]^ which can be detected through US by looking for at least one of the following characteristics: absence of interplate echo, irregular thickening of the inner margin of the craniosuture, or loss of the beveled margin.^[11]^ The utilization of 3D US in the diagnosis of craniosynostosis can prove advantageous, as transvaginal 3D US has the ability to more accurately identify fetal sagittal sutures, which are the predominant form of isolated craniosynostosis.^[12]^ Furthermore, 3D US can more accurately measure the width of the frontal suture and identify coronal suture deletion, allowing for earlier detection of Apert syndrome.^[13]^

### 4.3. Prenatal MRI findings

MRI is becoming more and more accepted as a component of the diagnosis for intrauterine microcephaly. However, there is still little agreement on the precise diagnostic standards or imaging parameters that can accurately determine the existence of microcephaly on MRI. Notwithstanding, there exist some frequently observed findings that are often linked with the condition. For example, a reduced complexity of cerebral gyri is a commonly encountered imaging feature. An MRI-based categorization method was proposed by Vermeulen et al in 2010 to determine if 12 children with a clinical diagnosis of microcephaly displayed a streamlined pattern of cerebral gyrification. To assess the degree of gyration complexity subjectively, they created a visual grading system with 3 points: normal, simple, and severely simplified. They also developed a scale to compare normal and pathological features in the basal ganglia and posterior fossa. The gyral index was used to do an objective morphological study on both scales, and the results were compared to normocephalic controls’ data.^[14]^ The research showed that compared to the normocephalic group, the microcephalic group had a much lower gyral index.^[14]^

Besides small head circumference, other abnormalities were detected in 76% of children with microcephaly through MRI examination, including white matter abnormalities (40%), corpus callosum abnormalities (31%), subtentorial lesions (15%), and cortical gyration abnormalities (14%). This suggests that MRI is a more sensitive imaging modality for identifying neurological lesions and microcephaly-related abnormalities.^[15]^ A large-sample, multi-center study conducted by Griffiths et al in 2017^[16]^ demonstrated that MRI improved the diagnostic accuracy of fetal intracranial abnormalities at 18 to 24 weeks of gestation by 23% (95% CI: 18%–27%) and at 24 weeks of gestation and above by 29% (95% CI: 23%–36%, *P* < .0001). The overall diagnostic accuracy of US and MRI was 68% and 93%, respectively, representing a 25% difference (95% CI: 21%–29%). MRI was found to provide additional diagnostic information in 49% of patients and alter prognostic information in at least 20% of cases. Moreover, it has led to changes in clinical management in more than one-third of cases. The optimal period for fetal MRI is between 28 and 32 weeks of gestation, as it provides the most comprehensive information about fetal brain anatomy. It is advised that fetal MRI be carried out in medical institutions equipped with specialists in fetal central nervous system imaging. MRI is typically not the primary diagnostic tool used for assessing fetal craniocerebral abnormalities. Instead, it is used as an additional examination following US. Moreover, due to factors such as claustrophobia and long detection times, it is generally not suitable for fetuses that require dynamic observation and repeated follow-up.^[1]^

MRI has the potential to assist in the prenatal detection of craniosynostosis. It can reveal simultaneous abnormalities in the fetal face and brain, as well as provide supplementary information. Although similar to US, MRI does not directly observe fetal cranial sutures, instead, it identifies potential cranial sutures through characteristic skull structures.^[17]^ In a study by Rubio et al,^[18]^ 6 cases of post-partum diagnosed craniosynostosis were reported, of which 5 cases were diagnosed only prenatally with the assistance of MRI. MRI can also detect abnormal features that may not be visible through US, such as agenesis of the corpus callosum, tetragonal cord, distal deformity, ophthalmia, and finger deformity. However, further research is needed to determine the value of MRI in the prenatal diagnosis of isolated microcephaly.

Additionally, the researchers found that there was a significant difference in brain volume between microcephalic patients and their normocephalic counterparts. Specifically, microcephalic patients were found to have reduced cerebral and cerebellar volumes at ages 1 to 2 years and 2.5 to 8.5 years. These findings suggest that MRI can enhance and potentially confirm the detection of microcephaly, as observed through US. They underscore the value of using both modalities in the overall diagnostic evaluation.

## 5. Conclusion

The diagnosis of prenatal fetal microcephaly requires careful consideration. When the fetal head circumference measures below −3 SD, abnormal fetal brain development should be considered. During fetal craniocerebral US at around 24 weeks of gestation, it is crucial to observe the clarity of the lateral fissure structure and the appearance of sulci gyri. If suspicious ventricle widening, small head circumference, or cerebellar dysplasia is observed, a detailed scan of the lateral fissure, parietal sulci, and taloccipital sulci is necessary to observe cortical thickness and morphology, in order to comprehensively evaluate the development level of the cerebral cortex. MRI is typically used to confirm fetal central nervous system malformations identified by US and to assess fetal brain development from multiple angles. It is widely considered the most accurate method for detecting fetal gyri and sulci abnormalities. However, caution must be exercised when evaluating developmental retardation of sulci and gyri using US, and dynamic observation must be conducted. When necessary, diagnosis should be confirmed with MRI.

## Acknowledgments

We thank Zhihui Xiong (Tongde Hospital of Zhejiang Province), for his assistance in the design of the research strategy and Guang Zhu (Tongde Hospital of Zhejiang Province), for his assistance with some of the graphical outputs of the results. Neither received any compensation for his contribution to the study.

## Author contributions

**Formal analysis:** Hai-lian Guan.

**Validation:** Feng Jin.

**Writing – original draft:** Shu-ping Pei.

**Writing –review & editing: S**hu-ping Pei.
